# Liquid crystal tunable claddings for polymer integrated optical waveguides

**DOI:** 10.3762/bjnano.10.209

**Published:** 2019-11-05

**Authors:** José Manuel Otón, Manuel Caño-García, Fernando Gordo, Eva Otón, Morten Andreas Geday, Xabier Quintana

**Affiliations:** 1 CEMDATIC, ETSI Telecomunicación, Universidad Politécnica de Madrid, Av. Complutense 30, 28040 Madrid, Spain; 2 INL International Iberian Nanotechnology Laboratory, Av. Mestre José Veiga s/n, 4715-330 Braga, Portugal; 3 Institute of Technical Physics, Wojskowa Akademia Techniczna, ul. Gen. Witolda Urbanowicza 2, 00-908 Warszawa, Poland

**Keywords:** liquid crystal, organic waveguide, photonic integrated circuit, polymer waveguide, tunable cladding

## Abstract

Optical waveguides in photonic integrated circuits are traditionally passive elements merely carrying optical signals from one point to another. These elements could contribute to the integrated circuit functionality if they were modulated either by variations of the core optical properties, or by using tunable claddings. In this work, the use of liquid crystals as electro-optically active claddings for driving integrated waveguides has been explored. Tunable waveguides have been modeled and fabricated using polymers. Optical functions such as variable coupling and optical switching have been demonstrated.

## Introduction

Photonic integrated circuits (PIC), also known as integrated optoelectronic devices, optical chips, planar lightwave circuits or integrated optical circuits [1], are microdevices that combine several photonic functions into the same chip. PICs are therefore considered the photonic equivalent to electronic integrated circuits (EICs), where signals are carried out by visible and near-infrared (NIR) photons rather than electrons. EICs have irreversibly modified our way of life through computers, communications and consumer electronics. Since their origin in 1954, EICs have been exponentially increasing the number of elements (transistors) according to Moore’s law [2]. A single chip of a current EIC accumulates billions (10^9^) of components.

PICs were born just 15 years later, in 1969 [3]. Notwithstanding the half century of development of the technology, the achievements are far more modest. The most complex PICs nowadays integrate a few thousand components at most. There are a number of reasons for this vast difference between the two technologies: (1) Photonic circuits, especially in the NIR range, are currently based on materials such as indium phosphide (InP), often requiring the use of ternary and quaternary materials for active components. (2) EIC components are interconnected by tiny nanometer-scaled lossless wires, while PIC components require interconnections with waveguides the size of which should be of about the same order as the working light wavelength. (3) EICs are made of a reduced set of simple building blocks –transistors, diodes, resistors, and capacitors– able to perform an amazingly large variety of functions. Such arrangement is not common for PICs yet [4].

Several approaches have been followed to overcome this problem. Some of these approaches rely on the upgrade of PIC waveguides, commonly passive, turning them into tunable devices able to drive and modulate light signals. One possible way that has attracted increasing interest in the last five years is the use of photonic circuits based on silicon. Silicon photonics can be employed in the NIR range at wavelengths longer than 1100 nm. This material paves the way to the use of large wafers, well-known efficient microelectronic processes and remarkable cost savings. Silicon waveguides can be developed on silicon dioxide, resulting in silicon-on-insulator (SOI) wafer structures compatible to CMOS processes [5]. This opens the possibility of integrating electronic and photonic components in the same circuit. The main drawback for silicon waveguides to become tunable devices is the big difference in refractive indices between the core (Si, *n* > 3.0) and the cladding (SiO_2_, *n* < 1.50). This difference confines the propagating wave inside the core, making the evanescent wave negligible [6]. In this situation, waveguide tuning based on core/cladding interactions is hindered. Nevertheless, silicon waveguides show a number of nonlinear optical effects [7], including a large thermo-optic coefficient that can be used in all-photon interactions within the photonic circuit [8].

A simpler approach for tunable waveguides could be the core/cladding interaction. Having an electro-optically active cladding would lead to waveguides the effective refractive index of which can be modified by external signals. However, this requires a reduced index gradient between core and cladding. In this work, the use of liquid crystals (LCs) as tunable claddings of waveguides has been explored. Given the range of refractive indices shown by LCs, the use of standard inorganic core materials is unfeasible. Instead, a number of organic cores have been tested.

## Results and Discussion

### Propagation in waveguide structures

**Figure 1:**
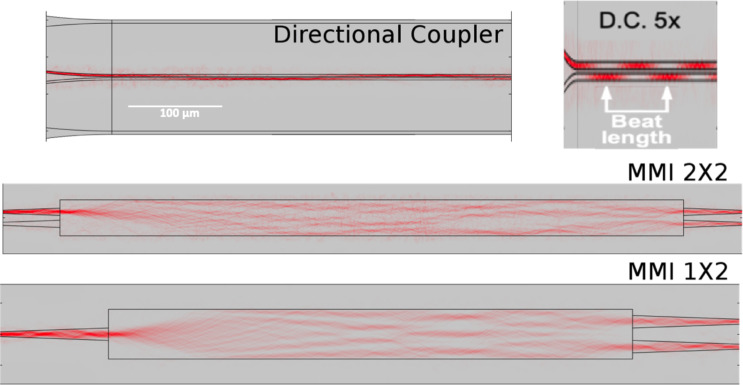
COMSOL simulations of light propagation in a directional coupler and two MMIs, all having rectangular cross sections. The red dots show the distribution of power density. The inset at the top right is the same sketch of the directional coupler, squeezed 5× horizontally and stretched 5× vertically to emphasize the power transfer and beat length.

Figure 1 depicts the electric field distribution in a directional coupler and two multimode interferometers (MMIs) with symmetric and asymmetric input, respectively. The optical power is split between the guides of the directional coupler, showing an alternating power transfer with a characteristic coupling distance called “beat length”, *L*_beat_.

The beat length depends on the difference between effective refractive indices of the odd and the even optical modes of the coupler [9]:

[1] $$L_{\text{beat}}=\frac{\lambda }{2\left(n_{\text{even}}-n_{\text{odd}}\right)},$$

and, thus, on the magnitude of the evanescent field and the distance between the guides. Practical uses of integrated directional couplers require the beat length to be in the micrometer range. This range is found either when the fraction of evanescent field is significant or when the distance between the guides is very low. The first option requires that core and cladding indices are quite close to each other; the second may often lead to complex manufacturing processes and results less manageable for preparing tunable devices. Polymer waveguides (PWGs) are well suited for preparing devices having LC cladding. Indeed, the refractive index of PWGs is typically in the range of 1.5–1.7, i.e., close to refractive indices of common LCs. This index matching would not be possible using the usual materials employed in inorganic waveguides, such as silicon or indium phosphide. Silicon nitride, with a refractive index of approximately 2, could be at reach of some high-birefringence LCs.

The same argument is true for MMIs. The lower part of Figure 1 shows the intensity distribution in a symmetric and in an asymmetric MMI. Power distribution varies with length, forming characteristic patterns with four, three, and two maxima at certain distances. These distances may vary under the influence of a tunable cladding; however, for the cladding to have a significant influence, a quasi-index matching between cladding and core is required. Again, the use of PWGs seems to be the most suitable option for the control with LC claddings.

### Modifying the beat length in directional couplers

**Figure 2:**
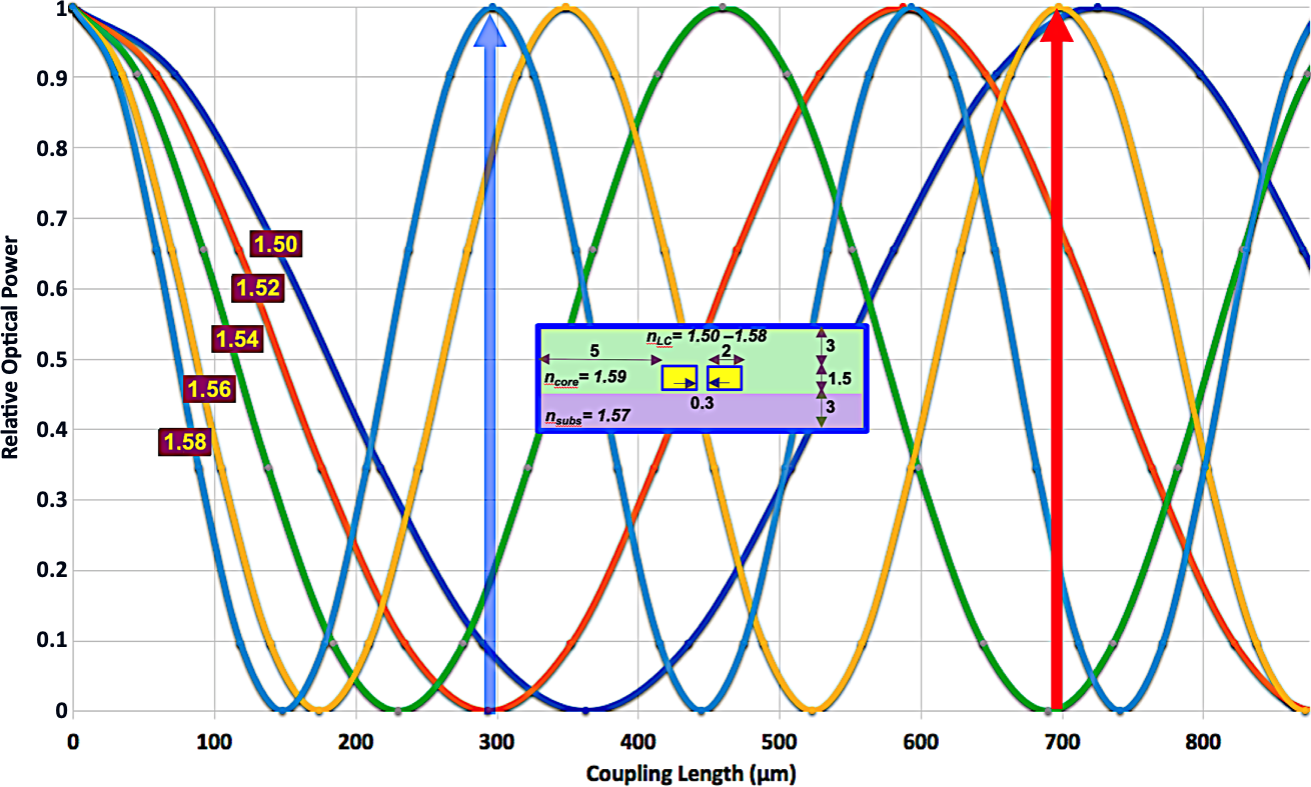
Power output in the input waveguide as a function of the effective refractive index of the LC cladding. The modeled directional coupler dimensions (in µm) and refractive indices are shown in the inset. The wavelength is 632.8 nm. The arrows show two examples of optical switches that could be obtained from this structure.

A simulation example of a directional coupler with an LC tunable cladding is shown in Figure 2. The dimensions and refractive indices, shown in the inset, have been chosen as typical values for organic PWGs and LCs. The curves show the effect of varying the LC effective refractive index from 1.50 to 1.58 in a structure the core of which has an refractive index of 1.59.

As seen in Figure 2, the cladding effect is significant. The beat length more than doubles upon varying the LC index over the mentioned range. Several practical devices could be prepared from this design, e.g., a variable coupler, the power ratio of which can be modified by external control, or an optical switch, such as those shown by the arrows, in which all the power is transferred to either output.

**Figure 3:**
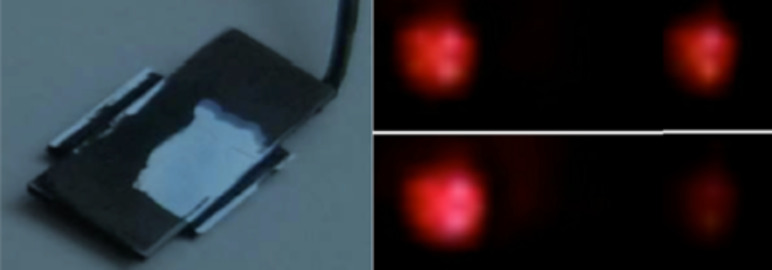
A directional coupler prototype showing power distribution between the output guides.

A practical realization of the above scheme is shown in Figure 3. The dimensions and refractive indices are similar to those in the simulation. The device (left) is fully painted in black to avoid spurious external light (a black area has been removed in the shown device). The coupler is tuned to switch between a 50:50 power distribution and (nearly) a 100:0 distribution.

### Modifying the optical length of MMIs

The functionality of multimode interferometers (MMIs) depends on their effective optical length. The effective optical length can be varied by either modifying the physical length or the effective refractive index. In the second case, one may modify the core index, as in devices exploiting the large thermo-optic coefficient of silicon [10] or the cladding index, as in the polymer guides with LC cladding studied here.

The effect of cladding on the MMI field profile is boosted by the increment of the evanescent field, which is achieved by reducing the physical dimensions of the waveguide, and the difference between the core and cladding indices. However, it is important to keep in mind that neither the dimensions nor the index differences can be arbitrarily reduced, since propagating modes may become leaky, and a significant fraction of the light power may be transferred to the cladding and dissipated. A trade-off between the need of close indices while minimizing leakage defines the MMI limits.

**Figure 4:**
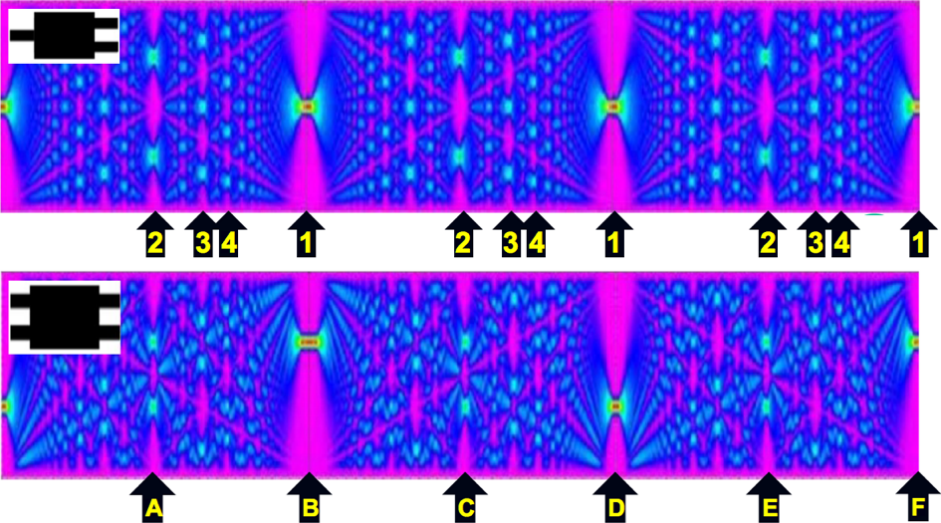
Power density distribution in the first three periods (3*L*_π_) of a 1 × 1 symmetric MMI and a 2 × 2 asymmetric MMI (insets, top left). The arrows in the top figure are effective device lengths where 2, 3, 4, and 1 outputs show up on each period (there are several solutions). The arrows in the bottom figure are effective lengths where the device behaves as a 50:50, a 100:0, and a 0:100 coupler. One can switch between these lengths modifying the refractive index of the LC cladding. This produces several tunable devices.

Figure 4 shows examples of MMIs with symmetric and asymmetric input. The light power distribution changes with the length of the device. Each mode in the MMI travels according to its specific effective refractive index and thus an optical power distribution pattern is generated from the interference of the modes present in the MMI. The pattern repeats at a certain distance [11] called the “characteristic length”, *L*_π_,

[2] $$L_{\pi }=\frac{\lambda }{2\left(n_{{\text{TE}}_{0}}-n_{{\text{TE}}_{1}}\right)},$$

similar to beat length of Equation 1, where the indices refer to the first two propagating modes. Depending on the setup, only TE or TM modes (seldomly both) couple to the waveguide. Assuming that only the first two modes propagate is fair, as long as the devices are designed with proper dimensions. Inside every characteristic length, patterns having several maxima can be seen. The distance *L_N_* at which these patterns shows up is

[3] $$\begin{array}{c} L_{N}=\frac{3L_{\pi }}{4N}\;\;\text{for symmetric MMIs}\text{, and}\\ L_{N}=\frac{L_{\pi }}{N}\;\;\text{for asymmetric MMIs}\text{,} \end{array}$$

where *N* is the number of maxima in the pattern.

Through modifying the characteristic length by switching the LC cladding, several tunable devices can be prepared. Since modes are characterized by a particular degree of confinement, every mode will be affected differently by a change in the cladding refractive index. For example, modifying the effective length from E to D or to F would change power splitting from 50:50 to 100:0 or 0:100, respectively. The same result would be obtained shifting from C to B or D. However, the manipulation of optical lengths with variable core or cladding refractive indices is constrained in the second case: The relative length variation from B to D is 100%, whereas going from D to F only requires 50% increment. When combined with the leakage issue mentioned above, the selection of tunable ranges within the MMIs becomes crucial.

### Determining the tunable range of LC-cladding MMIs

**Figure 5:**
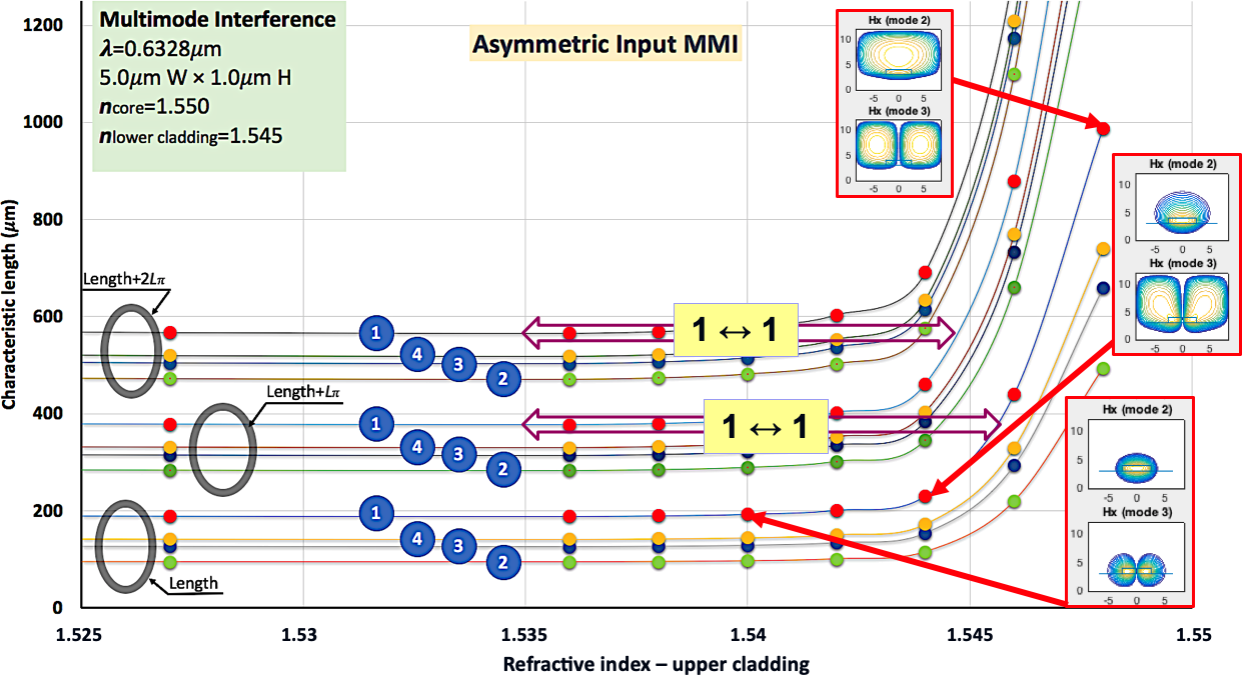
The characteristic length of an MMI is nearly constant until the cladding index reaches values close to the core index. Optical switches can be obtained in this region, as shown by the 1↔1 double arrows. However, modes become leaky when the cladding index values become too close to that of the core, as seen in the far-right insets.

The pattern seen in Figure 4 repeats itself giving a number of specific effective device lengths for which a cross section would lead to several well-defined output channels at different distances *L_N_*. A graph of the characteristic lengths for four different output configurations (green, blue, yellow, red for 2, 3, 4, 1 maxima) in three MMI periods (gray ovals) is shown in Figure 5.

This set of curves can be used for predictions of tenability of MMIs. By changing the refractive index from 1.535 to 1.544 (indicated by the upper {1↔1} range) one may switch the single output channels, corresponding to the positions D and F in Figure 4. A refractive index change from 1.535 to 1.546 could lead to a single channel switching corresponding to positions B and D.

The importance of targeting the lower refractive indices (as in the D-to-F transition) becomes evident when considering the confinement of the modes illustrated on the right of Figure 5. The top inset, corresponding to *n*_eff_ = 1.548, shows two leaky modes (actually they reach beyond the boundaries of the calculation). In other words, the refractive index of the cladding is too close to the refractive index of the core (1.550). The middle inset (*n*_eff_ = 1.544) shows one leaky mode while the other is confined with a large fraction of evanescent field. The lowest inset (1.540) shows two confined modes with a fair evanescent component. The waveguide cores are the small yellow rectangles in the lower part of each graph. These results demonstrate that devices such as optical switches may be feasible with this setup, assuming that leakage losses can be tolerated. The simulated MMI section is fairly short (ca. 250 µm); consequently, only minor losses shall be expected for modes not too far from guiding conditions. In any case, the working range appears to be quite strict; a careful design tailored for each set of parameters would be necessary.

**Figure 6:**
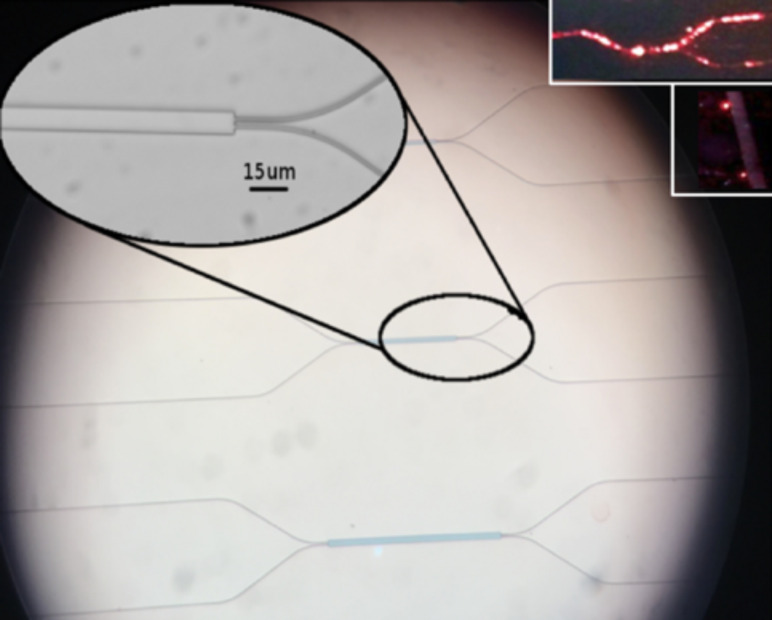
A number of SU8 PWG MMIs of different lengths prepared at the lab. The substrate is SiO_2_ on an Si wafer; the LC cladding is not deposited yet. Upper inset: MMI configured for a 100:0 coupling. Lower inset: outputs (about 95:5).

Figure 6 shows some examples of SU8 PWG MMIs prepared in the laboratory. The inset depicts a working MMI where light is being coupled to one of the inputs in a 100:0 configuration. The outputs are shown in the inset below (the actual power distribution is about 95:5, but the picture is saturated).

### LC-cladding of inorganic core MMIs

Confinement of radiation can also be achieved through increasing the core refractive index. Hence, calculations have been performed using an inorganic core of Si_3_N_4_, the refractive index of which is about 2.1. A hypothetical LC cladding working with this core would have to be highly birefringent, perhaps ∆*n* > 0.5. Although neither common nor commercially available, some experimental LC mixtures having such birefringence can be found in the literature [12].

**Figure 7:**
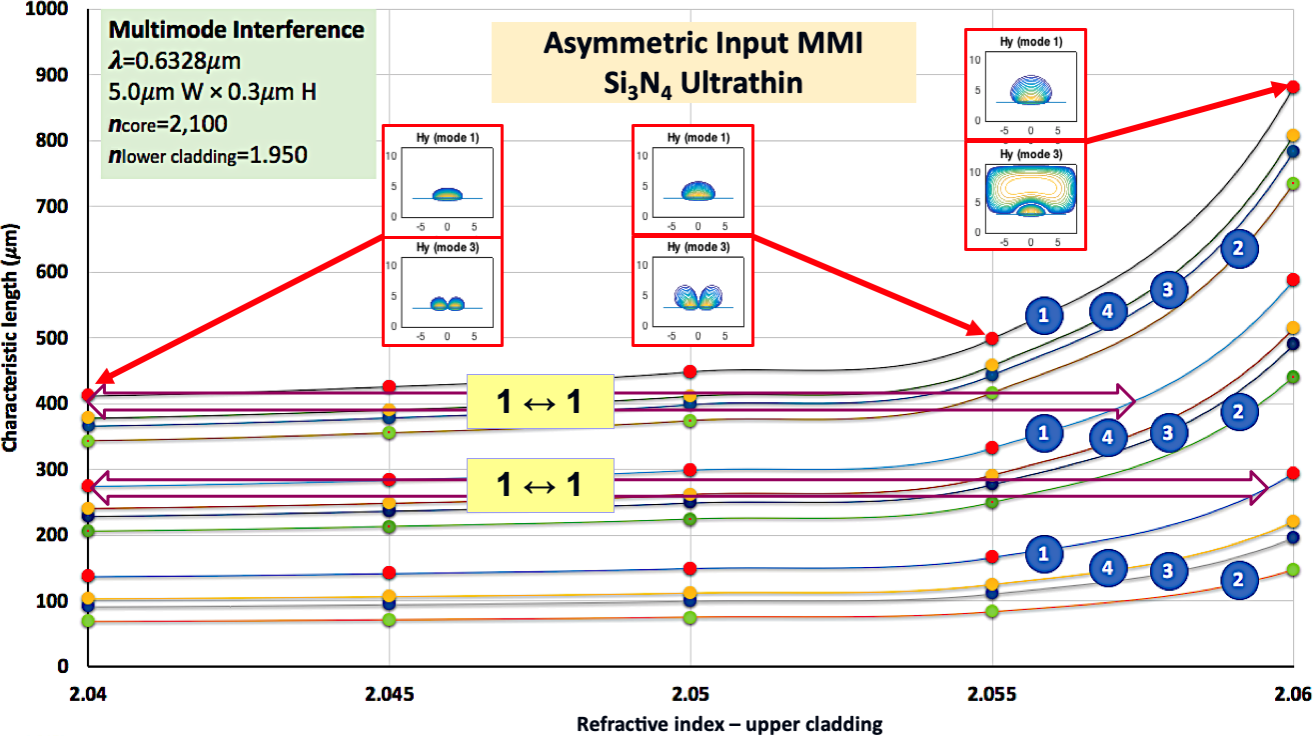
An MMI with inorganic Si_3_N_4_ core and high-birefringence LC cladding does not show variations in characteristic length, unless the waveguide thickness is significantly reduced. In this case, a working range for optical switches can be found.

Repeating the above calculation with waveguides having the same dimensions, and *n*_core_ = 2.10, the results vary considerably. The electric field is substantially confined to the core, with no leaks even for *n*_eff_ = 2.08 of the LC. The drawback is that the interaction between core and cladding is drastically reduced, and only switching {1↔2} (i.e., 100:0 to 50:50, or positions E and F in Figure 4) can be achieved for a device of 500 µm length. To overcome this situation, the fraction of evanescent field must be enhanced. This can be done by reducing the dimensions of the waveguide. Figure 7 shows the results obtained under the same conditions as in the previous calculations, except that the core height has been reduced to 0.3 µm. The refractive index of the LC cladding has been varied from 1.50 to nearly 2.10; in the Figure, the region of interest from *n*_eff_ = 2.04 to *n*_eff_ = 2.06 has been blown up. The radiation is well confined until about *n*_eff_ = 2.058, allowing for the definition of a fair working region for implementing optical switches.

## Conclusion

The extent of integration of photonic integrated circuits (PICs) is dramatically lower than that of electronic integrated circuits for a number of reasons. Developments fostering the PIC functionality without increasing its complexity are a possible way to contribute to new PIC realizations. Passive waveguides can contribute to managing guided optical signals by making them tunable using electrooptically active claddings. Liquid crystals can be used as active claddings in waveguides having a relatively low core refractive index. Simulations predict working ranges in which waveguides can be tuned to produce several devices, such as variable couplers and optical switches. Some of these devices have been implemented in polymer waveguides and their tunability has been demonstrated.

## Experimental

Several organic materials have been evaluated in a previous work [13]. The best results were obtained with direct laser writing (DLW) of UV-curing polymers specifically designed for optical waveguide manufacturing, such as the Ormocore/Ormoclad [14] and Epocore/Epoclad [15] families. Details of DLW manufacturing have been presented elsewhere [16].

**Figure 8:**
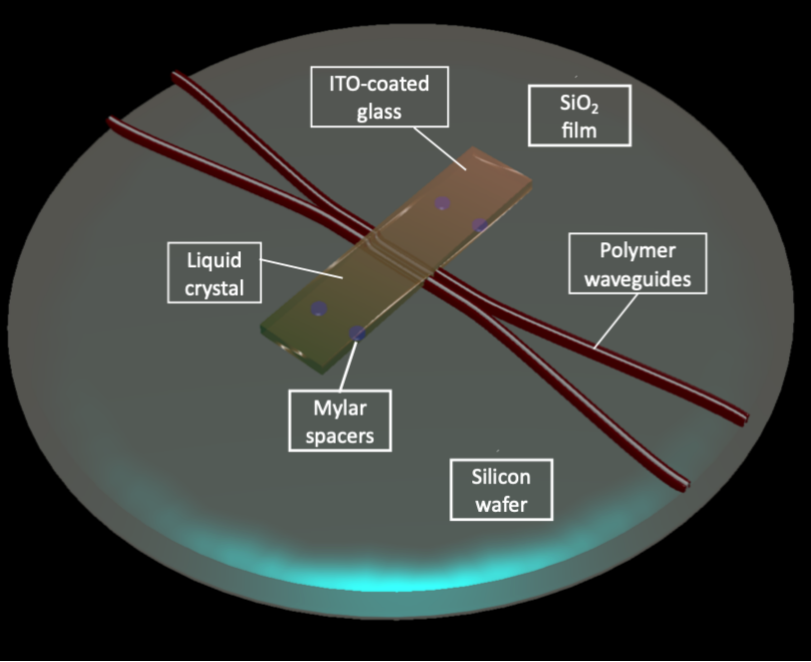
Two waveguides at short distance interacting through evanescent fields. The interaction is tuned by the liquid crystal acting as cladding.

A section of the waveguide, about 1 cm or less, was constructed as a directional coupler, consisting of two very close parallel waveguides without cladding, over which an LC cell was constructed. The cell consists of a LC (Merck MDA-98-1602, *n*_o_ = 1.52, *n*_e_ = 1.78) sandwiched between the substrate containing the waveguides and an ITO-coated glass plate to provide a conductive layer for applying electric signals (Figure 8). The substrate Si wafer was employed as counter electrode. Si was coated with approx. 4µm SiO_2_ (*n* ≈ 1.470) to avoid light leakage through the silicon for its refractive index is higher than the indices of the polymer. The LC layer thickness was 6 µm, controlled using a frame of Mylar spacers between the chip and the glass cover.

The inner glass plate surfaces were conditioned with spin-coated polyimide (PIA-2304, Lixon Aligner) for homogeneous LC alignment, parallel to the plate, along the waveguide. Both the glass surface and the waveguide were gently rubbed with a velvet cloth to induce the desired orientation. If an external electric field is applied to the LC structure with this alignment, one mode of light propagating through the waveguide would experience a cladding with a tunable refractive index in a range close to the LC birefringence ∆*n* = *n*_e_ − *n*_o_, where *n*_e_ and *n*_o_ are the extraordinary and the ordinary index of the LC, respectively.

In a second experimental setup, the two close waveguides of the directional coupler section were substituted by a single wider waveguide. The input and output waveguides were designed to be monomode at the working wavelength, while the wider section was multimode. The device is called multimode interference device (MMI). The same LC structure was mounted on top of the MMIs.

Modeling of mode propagation and electric field spatial distribution was performed employing the COMSOL^®^ finite element suite loaded with electromagnetics modules. The electric field distribution in waveguides normal sections was calculated with a code developed by the authors using MatLab-R2018b along with a number of MatLab scripts from the WGModes package [17] from the University of Maryland [18].
